# Labral Repair *Versus* Biceps Tenotomy/Tenodesis for the Treatment of Type II SLAP Lesions: Indications and Technique

**DOI:** 10.2174/1874325001812010269

**Published:** 2018-07-31

**Authors:** Chadwick C. Prodromos

**Affiliations:** Medical Director: The Illinois Orthopaedic Foundation, President, Illinois Sportsmedicine and Orthopaedic Centers ssistant Professor, Orthopaedic Surgery Rush University (Ret) 1714 Milwaukee AveGlenview IL 60025, USA; E-mails: chadprodromos@gmail.com

This issue contains articles by a distinguished group of shoulder surgeons on the treatment of Type 2 SLAP lesions. This is a controversial topic that largely revolves around the issues of:

- When to operate *versus* treat conservatively; and
- Whether to perform labral repair, biceps tenotomy/tenodesis, or both if surgery is deemed necessary

The keys to treating SLAP lesions for all surgeons are:

- proper diagnosis,
- an effective treatment algorithm, and
- safe effective surgical technique.

The majority of surgeons currently favor biceps tenodesis/tenotomy in older patients and labral repair in younger athletes. The case against labral repair is that it may not relieve pain and may result in stiffness. In contrast, I have favored repairing the labrum in nearly all cases and rarely if ever cutting the biceps. Our paper explains why we believe labral repair can result in consistently good results. It is because of this viewpoint that I was anxious to give a full hearing to those who do routinely cut the biceps.

Another interesting issue is whether to proceed with surgery at all in the patient with shoulder pain and a type two labral tear. In this regard, the piece by Dr. Schroeder is particularly enlightening [1]. Her landmark paper [2] found that sham surgery worked as well as labral repair, biceps tenotomy or biceps tenodesis. This is a truly amazing study and at a minimum should serve as a caution for anyone considering SLAP surgery with anything but crystal clear indications.

Imaging is another controversial area. Detachment of the biceps anchor, the essential lesion of the potentially operable type II SLAP lesions is particularly difficult to determine from imaging studies. The paper by Dr. Marder explains just what we can tell from advanced imaging studies [3].

This volume also has an international perspective with distinguished surgeons from around the world presenting their views. I hope that it serves its intended purpose of stimulating thought as well as providing guidance.
